# Sialic acid mediated transcriptional modulation of a highly conserved sialometabolism gene cluster in *Haemophilus influenzae *and its effect on virulence

**DOI:** 10.1186/1471-2180-10-48

**Published:** 2010-02-16

**Authors:** Gaynor A Jenkins, Marisol Figueira, Gaurav A Kumar, Wendy A Sweetman, Katherine Makepeace, Stephen I Pelton, Richard Moxon, Derek W Hood

**Affiliations:** 1 Molecular Infectious Diseases Group, University of Oxford Department of Paediatrics, Weatherall Institute of Molecular Medicine, John Radcliffe Hospital, Headington, Oxford, OX3 9DS, UK; 2 Maxwell Finland Laboratory for Infectious Diseases, Division of Pediatric Infectious Diseases, Boston University School of Medicine, Boston Medical Center, 774 Albany Street, Boston, MA 02118, USA

## Abstract

**Background:**

Sialic acid has been shown to be a major virulence determinant in the pathogenesis of otitis media caused by the bacterium *Haemophilus influenzae*. This study aimed to characterise the expression of genes required for the metabolism of sialic acid and to investigate the role of these genes in virulence.

**Results:**

Using qRT-PCR, we observed decreased transcriptional activity of genes within a cluster that are required for uptake and catabolism of 5-acetyl neuraminic acid (Neu5Ac), when bacteria were cultured in the presence of the sugar. We show that these uptake and catabolic genes, including a sialic acid regulatory gene (*siaR*), are highly conserved in the *H. influenzae *natural population. Mutant strains were constructed for seven of the nine genes and their influence upon LPS sialylation and resistance of the bacteria to the killing effect of normal human serum were assessed. Mutations in the Neu5Ac uptake (TRAP transporter) genes decreased virulence in the chinchilla model of otitis media, but the attenuation was strain dependent. In contrast, mutations in catabolism genes and genes regulating sialic acid metabolism (*siaR *and *crp*) did not attenuate virulence.

**Conclusion:**

The commensal and pathogenic behaviour of *H. influenzae *involves LPS sialylation that can be influenced by a complex regulatory interplay of sialometabolism genes.

## Background

Sialic acid (5-Acetylneuraminic acid, Neu5Ac) is a common sugar found as a terminal residue on glycoconjugates in many animals. In man, cell surface sialylation with Neu5Ac serves as a ligand for cell-cell adhesion, prevents complement activation and can help regulate tissue function and some cell signalling processes [1]. For *Haemophilus influenzae*, a Gram-negative bacterium found only in humans, the major surface glycolipid, lipopolysaccharide (LPS), can also be sialylated. This bacterium is an obligate commensal of the human respiratory tract but is able to cause significant disease. The majority of strains lack a capsule, so called non-typeable (NT*Hi*) strains, and commonly cause otitis media (OM), sinusitis and lower respiratory tract infections, and occasionally invasive disease. NT*Hi *LPS plays a role in the complex interactions with the host required in both its commensal and pathogenic behaviours. Sialylation of LPS is a relatively common structural modification among mucosal pathogens such as *H. influenzae*, with a reported role in virulence in a number of organisms. LPS sialylation influences the resistance of *H. influenzae *to the killing effects of normal human serum as evidenced by decreased survival in normal human serum of sialylation-deficient mutants, for example those in which the CMP-Neu5Ac synthetase gene (*siaB*) has been disrupted [2]. Moreover, the *in vivo *role of Neu5Ac as a critical virulence factor in the pathogenesis of experimental OM has been demonstrated as Neu5Ac-deficient mutants were profoundly attenuated in animal models [3,4]. Sialylation of LPS interferes with the binding and activation of complement components of the host immune system on the bacterial surface [5]. Further, a role for LPS sialylation in 'biofilm' formation has been proposed that may be relevant to both the commensal behaviour and virulence of NT*Hi *[4,6,7].

**Figure 1:**
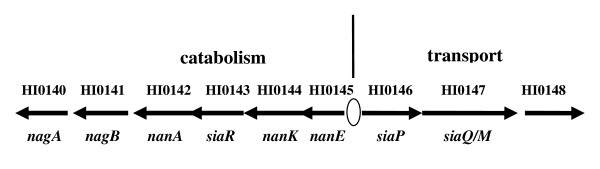
**The sialometabolism gene cluster of *H. influenzae***. Indicated are the catabolism and transport groups of genes, each gene is represented by an arrow indicating the direction of transcription. The HI numbers corresponding to the reading frame designation in the strain Rd genome sequence are given above the arrows and the gene names below. 0 indicates the position of the CRP binding sequence.

*H. influenzae *cannot synthesize Neu5Ac *de novo *[8] and, *in vivo*, NT*Hi *scavenges Neu5Ac from the host [3]. Neu5Ac is thought to be present at levels of about 0.5 mg/ml in human serum [8] and in addition to being incorporated into LPS, Neu5Ac may also be used as a carbon and energy source [9]. Bioinformatic analysis has shown that the key genes required for the dissimulation of Neu5Ac are present in *H. influenzae *[8] and recent studies have identified a high affinity TRAP (**Tr**ipartite **A**TP independent **P**eriplasmic) transport system encoded by the genes *siaP *and *siaQM *as the main uptake system of NT*Hi *for procuring Neu5Ac [10,11]. The genes for sialic acid catabolism and procurement are contiguous on the *H. influenzae *genome [8,12] and are arranged as two divergently transcribed operons (Figure 1). These nine genes are referred to as the sialometabolism gene cluster. The mechanism for regulation of *H. influenzae *sialic acid utilisation, whereby the entry of Neu5Ac into the catabolic pathway and incorporation in LPS is coordinated, is complex [12]. Located within the catabolic genes is *siaR*, encoding a protein containing two domains (helix-turn-helix and sugar isomerase) associated with sugar metabolism and regulation [13,14], that acts as a repressor of sialometabolism genes [12]. cAMP receptor protein (CRP) has also been shown to regulate the expression of the sialic acid uptake but not the catabolic genes [12].

In the present study we used reverse transcriptase PCR to investigate sialometabolism gene transcription in *H. influenzae *wild type and sialometabolism mutant strains following growth of bacteria in the presence or absence of added sialic acid. Strains mutated in sialometabolism genes have been investigated in *in vitro *and *in vivo *assays and a complex process of regulation of Neu5Ac metabolism has been confirmed.

## Methods

### Strains and culture conditions

*H. influenzae *strain RM118 (Rd) is a capsule deficient derivative from a serotype d strain for which the complete genome sequence has been obtained [15]. NT*Hi *isolates used in this study are representative of the genetic diversity of *H. influenzae *[16], and have been reported previously [17]. *H. influenzae *was grown at 37°C in brain heart infusion (BHI) broth supplemented with 10 μg haemin ml^-1 ^and 2 μg NAD ml^-1^. BHI plates were prepared with 1% agar and supplemented with 10% (v/v) Levinthals base. For selection following transformation, 10 μg kanamycin ml^-1 ^was added to the medium. For some experimental growth of *H. influenzae *we used chemically defined medium (CDM) [18]. When appropriate, Neu5Ac was added at 25 μg ml^-1 ^(BHI) or 30 μg ml^-1 ^(CDM) to the medium. *Escherichia coli *strain DH5α was used to propagate plasmids and was grown at 37°C in LB broth [19] supplemented when appropriate with 100 μg ampicillin ml^-1 ^or 50 μg kanamycin ml^-1^.

### Construction of *H. influenzae *mutant strains

The cloning and inactivation of *siaP *(*HI0146*), *siaQ/M *(*HI0147*) and *HI0148 *have been previously described [10]. Mutations were engineered in genes (*HI0142-HI0145*) and in *crp *by the following general method; the gene of interest was first amplified by PCR using locus specific primers (listed in Table 1) and strain Rd chromosomal DNA as the template under conditions described previously [20]. Amplification products were ligated into PCR cloning vectors pT7Blue (Novagen) or pTOPO (Invitrogen) and transformed into *E. coli*. Correct clones were confirmed by restriction analysis and PCR amplification. Genes were inactivated by ligating the kanamycin resistance cassette (kan^R^), from pUC4Kan, into suitable restriction sites within the reading frame. kan^R ^does not prevent transcriptional read through when in the same orientation as the target gene. When cloning into the pTOPO plasmid, kan^R ^present in the cloning vector was inactivated by digestion with NcoI and end-filling of the DNA ends with Klenow enzyme and dNTPs. Following re-ligation the plasmid was transformed into *E. coli *DH5α. Genes *HI0144 *(*nanK*) and *HI0145 *(*nanE*) were amplified together using the primers 0145for and 0143rev (Table 1) and each gene was then inactivated independently by insertion of kan^R ^at NruI and BglII sites respectively. For *nanA *(*HI0142*), insertion of kan^R ^was achieved following partial digestion with Mfe1 and *siaR *(*HI0143*) was inactivated by inserting kan^R ^at an MfeI site.

**Table 1:** Oligonucleotide primers used in this study.

primer	Sequence (5'-3')		primer	Sequence (5'-3')
0140for	CTGCAATTAAATGGCTGTGG		0140rev	GCAATTGTGTCATTCGCATC

0141for	TCAGTTGTTGGGCTGCAC		0141rev	CAGCAACTGCGCCTTCTA

nanAfor	TCCGCCATAATATCGACAAA		nanArev	TTTGCTTTTGCAAGCTGTTC

0143 for	AATTGCCGATACGATTTTGC		0143rev	TATCTTCTTCGCCCTGCACT

0144for	TGCGTTGTTTAGCACTAG		0144rev	GCTAATCCCACACTGCCA

0145 for	TTGCCAACCTGTCGATGA		0145rev	CCCTCAGCCATCACAAAACA

0146for	TGTTCTTGCCGCTGATTATG		0146rev	CATTTTCGGCAGCATCTTTT

0147for	GGAGTGAAGAACTCGCCAAC		0147rev	TCACGCATTGCTTTGATTT

0148for	TTTTTCAGCGAACGCACA		0148rev	TCAGTTTCACCGCCAATCA

FRDL	CCCTCAATTTGGTTTAAATCCTG		FRDR	CCATGGTCACGGTTATCAAGA

HI1045L	CAAGAAGTGCTTTCTCAAATTCAA		HI0145R	TTTATCCATTGGGCCATCAT

HI0146L	TCTGACTTTACCTTTGCAGAAT		HI0146R	AATACTGCCGCTTCAGGGTA

HI0143L	AAATCGCAAAACAAAATGGTG		HI0143R	CGGGGGAACGCAAACTAT

crpA	GCAACTCAACGAGATCCC		crpD	GACCAATCCTGTCTTCCT

nagE	GAACCGCCCACATATAAG		nagF	TGCGTTGTTTAGCACTAG

Mutant *H. influenzae *strains were constructed following transformation [21] of strain RM118, NT*Hi *375 or 486 using the appropriate plasmids that had been linearized by restriction endonuclease digestion. The resulting mutant strains were confirmed as correct after growth on BHI/kanamycin and by both PCR and restriction digestion analyses.

### Analysis of LPS by electrophoresis

Bacterial lysates were prepared from cells grown overnight on BHI plates to which Neu5Ac had been added. Lysates were then analyzed by tricine-SDS-PAGE and staining with silver as described previously [22].

### Serum bactericidal assay

Bacteria cultured on BHI plates to which Neu5Ac has been added were assayed for killing by pooled human serum, as described previously [2].

### RT-PCR analysis

Bacteria were cultured in BHI or CDM medium, with or without added Neu5Ac. When the OD_600 _reached 0.3 (CDM) or 0.6 (BHI), 1 ml aliquots of cells were collected and added directly to 2 ml RNA Protect Bacterial Reagent (Qiagen) and RNA was extracted using a SV Total RNA Isolation Kit (Promega). cDNA was synthesized by adding approximately 500 ng RNA, quantified by a Nanodrop spectrophotometer, to 2 μl Random Primers (Promega, 500 μg ml^-1^), l μl RNasin (Promega, 40 U μl^-1^), and the volume made up to 12 μl with DEPC-treated water. After heating at 70°C for 10 mins the sample was cooled on ice and a 1 μl aliquot removed to be used in a control PCR to ensure that the sample was DNA free. A mix of 4 μl DEPC water, 5 μl of 5× Buffer (Invitrogen), 1 μl dNTP's (25 mM Invitrogen), 2 μl of 0.1 M DTT (Invitrogen) and 1 μl M-MLV-Reverse Transcriptase (Invitrogen, 200 U μl^-1^) was added to the reaction and incubated at 37°C for 1 hour followed by 95°C for 5 mins. 1 μl of cDNA was then used as template in subsequent PCR reactions (RT-PCR), carried out using the conditions described above, or in real-time quantitative PCR (q-PCR). q-PCR reactions were performed in triplicate using the Corbett Research Rotor Gene RG-3000. Each reaction was performed in an individual tube and made up to 25 μl containing 5 μl cDNA, 12.5 μl PCR Master Mix (Abgene), 0.25 μl probe, 1 μl of forward and reverse primer and 5.25 μl H_2_O. Conditions for the q-PCR reaction were 2 min at 50°C, 10 min at 95°C and then 40 cycles, each consisting of 15 s at 95°C, and 1 min at 60°C. The housekeeping gene, *frdB*, was used as the reference gene. Left (L) and Right (R) primer pairs for genes *frdB, siaR, nanE *and *siaP *are given in Table 1. Probe #s 3, 59, 137 and 59 (Roche) were used respectively in the q-PCR reactions for these genes. Relative quantitation of gene expression was performed using the method described by Pfaffl [23]. Results given are based on the mean value of PCRs performed in triplicate in the same experiment. q-PCR was repeated a minimum of three times for each gene using independent cDNA and mRNA preparations from different batch growths of bacteria.

### Chinchilla model of Otitis Media

An experimental chinchilla (*Chinchilla lanigera*) model of acute OM was used [24]. Animal care and all related procedures were performed in accordance with institutional and federal guidelines and were conducted under an Institution Animal Care and Use Committee-approved protocol at Boston University Medical Centre [3]. Wild type NT*Hi *375, 486 and RM118 and their respective isogenic mutant strains (*nanA, siaR, siaP, crp*) were grown overnight for 16 hours in BHI broth. For animal challenge, the overnight grown bacteria were diluted in Hank's balanced salt solution (HBSS) and approximately 50-100 c.f.u. in 100 μl were inoculated through the left superior bulla of adult chinchillas with a 25-gauge tuberculin needle [3,5]. After seventy-two hours, tympanometry, otomicroscopy, and middle ear cultures were performed to determine if infection was present. The middle ear cavity was accessed and a direct culture was obtained as described previously [5,24]. Middle ear fluid (MEF) when present was obtained and if MEF was absent the middle ear was flushed with HBSS, 10-fold serial dilutions were prepared as previously described [3,5]. A volume of 100 μl of each dilution was plated onto chocolate agar plates (Remel). The lower limit of detection of viable organisms in MEF using this dilution series is 100 c.f.u. ml^-1 ^[3]. Direct and indirect examination of the ears was performed on days 3, 7, 12, and 19 following inoculation with NT*Hi *strains, and days 3, 7 and 11 following inoculation with strain Rd, or until the middle ear cultures were sterile on two consecutive samples. The median bacterial density was calculated for each organism at each sample point and statistically significant differences were determined using the Wilcoxon Rank-Sum Test (SaS**^®^9)**.

## Results

### The genes of the sialometabolism region are conserved in *H. influenzae*

Previous studies by us using a *H. influenzae *whole genome microarray [25] and by others [12] identified a region of DNA comprising nine contiguous genes that encode functions relating to sialometabolism (Figure 1). The genes for sialic acid catabolism (*HI0140 *(*nagA*), *HI0141 *(*nagB*), *HI0142 *(*nanA*), *HI0144 *(*nanK*), *HI0145 *(*nanE*) and including *HI0143 *(*siaR*)) and procurement (*HI0146 *(*siaP*), *HI1047 *(*siaQM*), *HI0148*) are transcribed divergently (Figure 1). *siaR *and *nanK *possess overlapping ORFs whilst three pairs of adjacent genes have intergenic regions of <50 bp.

To explore how general this arrangement of the sialometabolism region of DNA is in *H. influenzae*, we examined 25 NT*Hi *isolates selected because they are epidemiologically distinct and representative of NT*Hi *genetic diversity [17]. All 25 isolates incorporate sialic acid into their LPS as a terminal residue [26], although the location and amount of Neu5Ac in LPS glycoforms, and the repertoire of sialyltransferase genes present, are variable between strains. PCR analysis was carried out on chromosomal DNA from each strain using internal primers for each of the 9 genes (*HI0140-HI0148*) (Table 1) and primers designed against genes in the flanking regions (*HI0139 *encoding P2 protein on the 5' side and *HI0148.1/HI0149 *on the 3' side). This analysis confirmed that both the presence of individual sialometabolism genes and their organization in all 25 NT*Hi *strains was conserved and overall was the same as that of strain Rd (Figure 1). *H. influenzae *type b strains also maintained the sialometabolism gene cluster (data not shown). Two of the twenty five NT*Hi *strains, 375 and 486, which have been used in previous *in vitro *and *in vivo *studies of sialic acid metabolism, were selected for further investigation together with strain Rd. Mutations in genes within the sialometabolism region of DNA in strains Rd, 375 and 486, with the exception of *nagA *and *nagB*, were made. *nagB *encodes the last of the five steps of the Neu5Ac catabolic pathway (converting glucosamine-6-phosphate to fructose-6-phosphate), suggesting that the gene product may be essential because of its close association with central metabolism, as had been previously described for *nagA *[27].

### The sialometabolism uptake genes are essential for LPS sialylation and virulence

**Figure 2:**
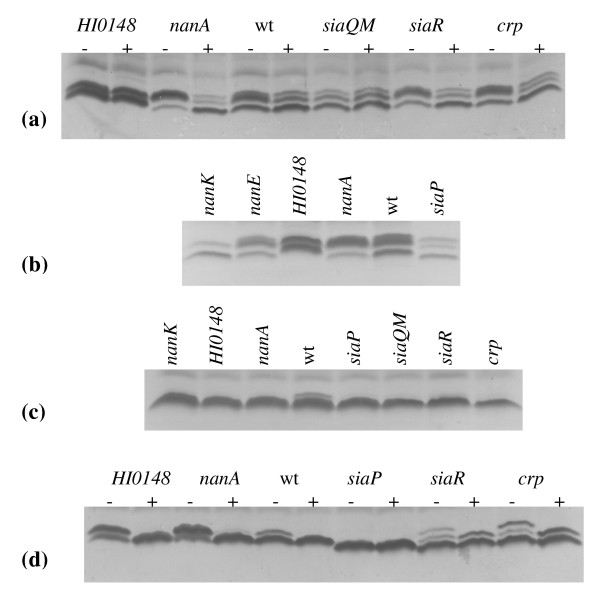
**T-SDS-PAGE analyses of LPS isolated from wild type (wt) strains Rd, 375 and 486 and their respective mutants**. Panels (a) and (d) show profiles of LPS without (-) and with (+) neuraminidase treatment. The wt or mutant strains are indicated above each lane. Shown are: panels (a) and (b), strain Rd; panel (c), strain 375; panel (d), strain 486.

*H. influenzae *possesses two genes,* siaP *(*HI0146*) and *siaQ/M *(*HI0147*) separated by 60 bp, which encode a two component TRAP transport system [10,11]. The phenotypic effect of mutation of *siaP *and *siaQ/M *on LPS structure of NT*Hi *strains was analyzed using gel electrophoresis. In agreement with previous studies using strain Rd [10] and NT*Hi *2019 [12], *siaP *and *siaQ/M *mutants of NT*Hi *strains 375 and 486 showed altered mobility of LPS consistent with a loss of sialylated LPS glycoforms when compared to the respective wild type (Figure 2). Further, the *siaP *mutant of strain 486 showed no change in LPS profile upon neuraminidase treatment (Figure 2). These data are fully consistent with the TRAP transporter being the primary means of sialic acid uptake in these NT*Hi *strains.

**Figure 3:**
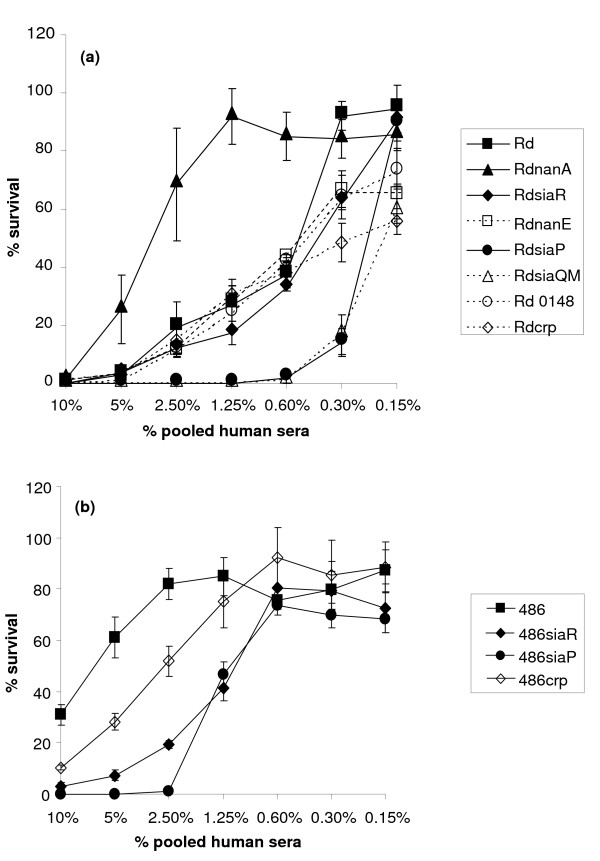
**Resistance (% survival) of *H. influenzae *strains to the killing effect of normal human serum**. 500 organisms of strain Rd (panel a) or NT*Hi *486 (panel b) or derived mutants were added to different (doubling) dilutions of pooled human serum; percentage survival of inoculum of bacteria (y-axis) is shown for varying serum concentrations (x-axis). Each point is the averaged result of 3 independently performed experiments, error bars (1 standard deviation) are shown.

Sialylation of LPS [28] is known to be an important virulence factor in *H. influenzae*, conferring increased resistance to killing by normal human serum [2,3]. There was a marked decrease in the survival of mutants deficient in sialic acid uptake compared to wild type for strains Rd (Figure 3a), 486 (Figure 3b) and 375 (data not shown) following exposure to pooled human serum for 45 mins, in agreement with previously published data [10].

By comparison, for strain Rd, the phenotype of a Rd*nanE *mutant, affected in Neu5Ac catabolism, was relatively unchanged compared to wild type based on electrophoresis of LPS (Figure 2b) and susceptibility to killing in a bactericidal assay (Figure 3b). However, when a Rd*nanA *mutant was compared to wild type by SDS-PAGE it was hypersialylated (Figure 2a) and showed increased serum resistance to killing when compared to the parent strain (Figure 3a). The changes in LPS profile when comparing the wild-type to strains with mutations in sialic acid catabolism genes in the 486 and 375 backgrounds were generally similar to the changes observed for strain Rd (data not shown).

**Figure 4:**
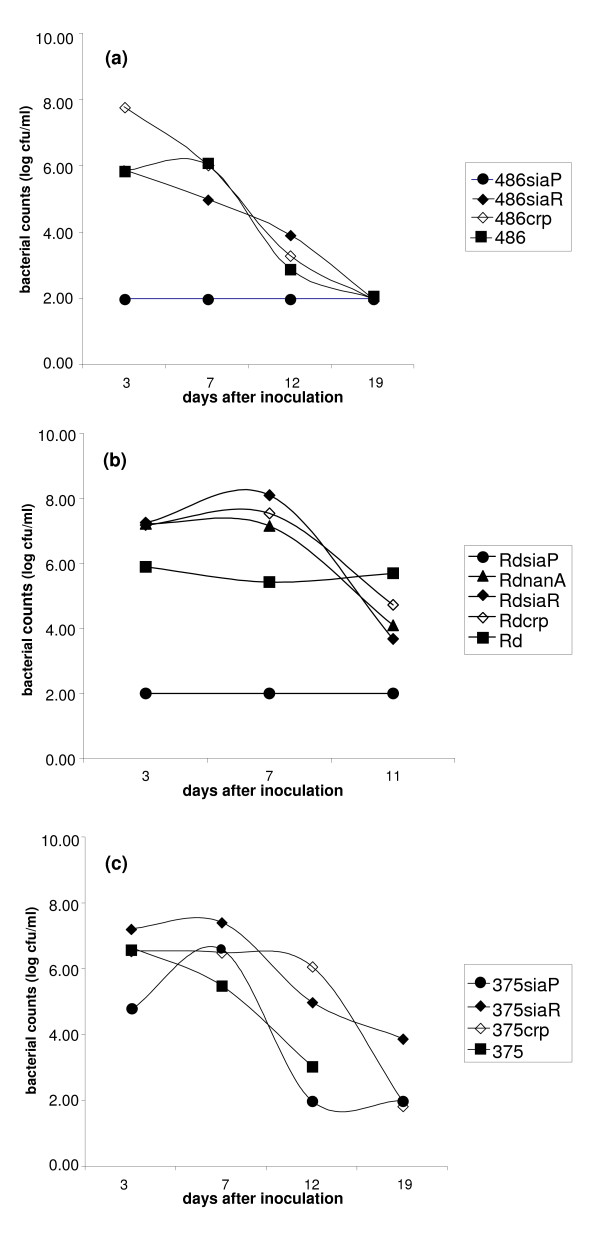
**Effect of mutation of *siaP*, *siaR *and *crp *on bacterial counts of *H. influenzae *strains from the middle ear of chinchillas when compared to wild type strains**. Animals were inoculated with between 60 and 100 organisms directly into the middle ear bullae. Each data point represents the average number of organisms ml^-1 ^of exudate or washings from the middle ear for typically four animals at different times (days) following inoculation. Shown are wild type and isogenic strains for: panel (a), NT*Hi *486; panel (b), Rd; panel (c), NT*Hi *375. The lower detection limit is a bacterial count of 2.00.

NT*Hi *strains 375 and 486 have previously been used to investigate the role of sialic acid as a virulence factor in a well described chinchilla model of OM [3,5]. For NT*Hi *strains 375, 486 and strain Rd, we compared wild type and *siaP *mutants; approximately 100 c.f.u. were inoculated directly into the bullae of four animals in each group, with the exception of 375 wild type (2 animals). For strains Rd and 486, *siaP *mutants with a deficient TRAP transport system were clearly attenuated, with low or undetectable bacterial counts in the middle ear after two days (Figure 4). All middle ears (100%) inoculated with strains 486 and Rd developed high-density infection compared to the absence of middle ear disease in animals challenged with *siaP *mutants; 486*siaP *(0/4 ears culture positive; p = 0.02), Rd*siaP *(0/4 ears culture positive; p = 0.03). For strain 375, the attenuation was less marked (Figure 4) and not statistically significant for the *siaP *mutant compared to the wild-type strain (375*siaP *3/6 ears culture positive; p = 0.39, but sample for 375 wild type was from only 2 animals). This is possibly due to the low levels of LPS sialylation observed for strain 375. Strain Rd*nanA *which showed enhanced LPS sialylation *in vitro *was of equivalent virulence to the parent strain in the middle ear of the chinchilla (Figure 4) (no statistically significant difference between Rd and Rd*nanA *(4/4 ears culture positive; p = 0.31)).

### Sialylation of *H. influenzae *LPS is adaptive and is subject to complex regulation

Sialic acid may be incorporated into LPS or utilized as a source of carbon and nitrogen for NT*Hi*. In the host, given the context of the complex array of other potential nutrients available to *H. influenzae *and the two potential fates for Neu5Ac in the bacterium, it is reasonable to assume that sugar utilization in *H. influenzae *is regulated at the genetic level. The intervening 353 bp between the sets of divergently transcribed sialometabolism genes include the binding sites for the regulatory proteins SiaR and CRP [12].

In our experiments, mutation of *siaR *showed somewhat different phenotypes dependent upon the strain background. Compared to wild type, the Rd*siaR *mutant strain showed little difference in LPS phenotype (Figure 2d), but was slightly more susceptible to killing in the serum bactericidal assay following growth in the presence of added exogenous sialic acid (Figure 3a). A reduction of serum resistance of a 486*siaR *mutant (Figure 3b) compared to the parent strain is consistent with some LPS truncation (Figure 2d), although the reason for this is unknown. No significant difference between strains 375 and 375*siaR *was seen for LPS phenotype (Figure 2c) or in the bactericidal assay (data not shown). Importantly, in the chinchilla model of OM, mutation of *siaR *in strains Rd, 375 and 486 produced strains that were virulent (Figure 4), although we cannot rule out some difference in bacterial titres during the course of disease. Thus, *siaR *is not essential for virulence in this model.

There is a consensus sequence for CRP binding (**TGTGA**TCAACT**TCTCA**) within the DNA region intergenic between *nanE *and *siaP *[12,29], consistent with the role of CRP in regulating Neu5Ac uptake genes. Of the mutant strains with *crp *inactivated, only NT*Hi *486 displayed any alteration in LPS profile (Figure 2d) and some increased serum sensitivity compared to the parent strain (Figure 3b). Significantly, *in vivo *in the chinchilla, each of the strains Rd*crp*, 375*crp *and 486*crp *were virulent (Figure 4).

**Figure 5:**
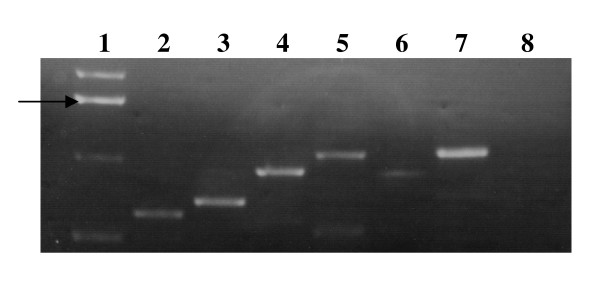
**PCR amplification for cDNA of sialometabolism genes from strain Rd showing co-transcripts for adjacent gene pairs**. cDNA was made after bacteria were grown in BHI in the presence of sialic acid. RT-PCR products shown are in lane 2, *nagA*/*nagB*; lane 3, *nagB*/*nanA*; lane 4, *nanA*/*siaR*; lane 5, *siaR*/*nanK*; lane 6, *nanK*/*nanE*; lane 7, *siaP*/*siaQM*; lane 8, *siaQM*/*HI0148*. Lane 1 shows the 1 kb DNA ladder marker with the 1.6 kb band marked by an arrow.

To investigate in more detail the interdependence of genes involved in sialometabolism, we compared gene expression in wild type and mutant strains following growth in the presence or absence of exogenous Neu5Ac. RT-PCR analysis of total RNA extracted from strain Rd mutated in each of the genes *nanA, siaR, nanK, nanE, siaP, siaQM, HI0148 *and *crp *was performed using internal pairs of primers specific for each gene of interest (Table 1) and the levels of expression compared using the RT-PCR amplification product for the housekeeping gene, *frdB*, as a control between samples. The level of transcript for each sialometabolism gene was generally greater in the *siaR *mutant background when compared to the wild type strain, although the results proved difficult to quantify (data not shown). This would be consistent with SiaR exerting a regulatory (negative) effect on sialometabolism gene expression, i.e. acting as a repressor [12]. The corresponding change in expression of multiple genes might suggest some co-regulation or co-dependence. Using primer pairs targeted against the 5' and 3' ends of adjacent genes across the region, RT-PCR analysis showed some co-transcripts for most gene pairs across the sialometabolism region (Figure 5).

**Figure 6:**
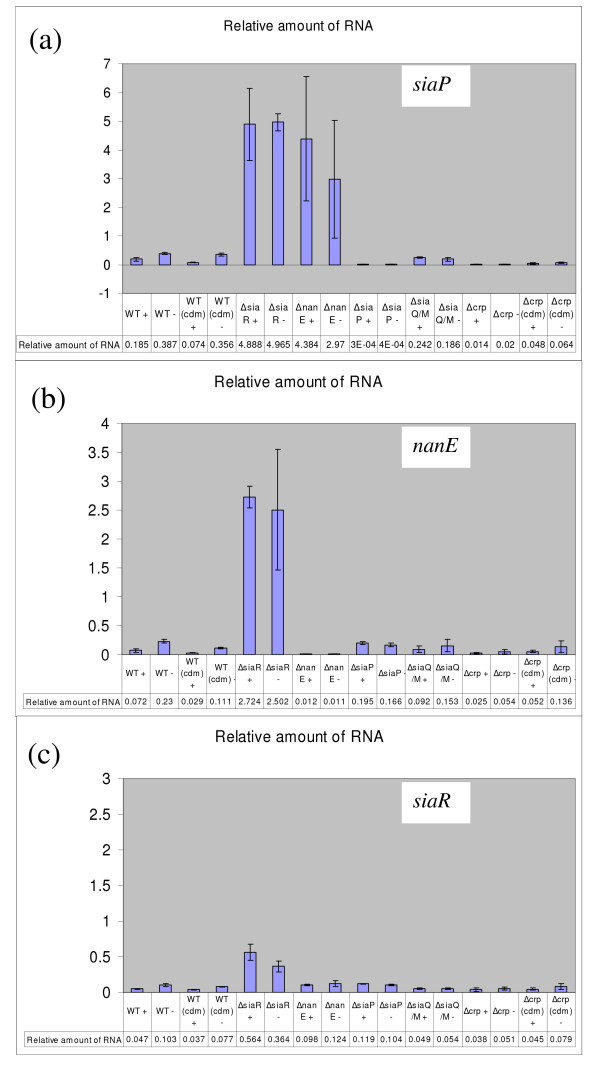
**q-PCR data for sialometabolism genes of *H. influenzae***. In each panel, the y-axis shows the quantity of mRNA, relative to the *frdB *control gene, for cDNA from wild type or mutant strains following growth in the presence (+) or absence (-) of exogenous Neu5Ac (x-axis). Shown are: panel (a) *siaP*; panel (b) *nanE*; panel (c) *siaR*. Each value shown below the x axis represents the results from 3 separate experiments utilising independent cDNA and mRNA preparations and each q-PCR reaction was run in triplicate. The error bars indicate the standard deviations derived for the respective data.

We obtained quantitative data for the changes in the level of expression of representative sialometabolism genes (*siaR, nanE, siaP, HI0148*) by q-PCR. These data confirmed the key observation from our initial microarray experiment [25], i.e. that there is a small but consistent two to three fold repression of sialometabolism gene expression following growth of strain Rd in the presence of exogenous sialic acid in both BHI and the chemically defined (CDM) media (Figure 6, Table 2). Quantification of the changes in transcript levels of the first gene of each of the divergently transcribed sialometabolism regions *nanE *(catabolic) and *siaP *(transport) in the *siaR *mutant background showed 11 and 13 fold increased expression levels respectively when compared to the parent strain following growth in the absence of added Neu5Ac (Figure 6) confirming that SiaR acts to repress both the catabolic and uptake genes. Changes in gene expression in response to exogenous Neu5Ac, however, were not evident in the *siaR *mutant strain (Figure 6) although *siaR *expression was itself slightly repressed (2 fold) following growth of the wild type strain in the presence of sialic acid. A transcript for the *siaR *gene was unexpectedly detected from the *siaR *mutant strain in both our q-PCR and RT-PCR experiments; in the latter, the size corresponded to that of the native gene. DNA sequencing of this cDNA revealed that kan^R ^had been deleted leaving a 1 bp insertion that constituted a frame-shift of the *siaR *ORF. The reason for the apparent instability of kan^R ^in this gene following reverse transcription is not understood. The *siaP *gene showed a significant 8 fold increase in expression in the *nanE *mutant strain compared to the parent strain, following growth without added Neu5Ac (Figure 6).

**Table 2:** Transcription analyses of sialometabolism genes in Rd and derived mutant strains.

	Gene expression ratio:		
	
strain	*siaP*	*nanE*	*siaR*
**Rd**	2.1	3.2	2.2

***siaR***	1.0	0.9	-

***nanE***	0.7	-	1.3

***siaP***	-	0.9	0.9

***siaQ/M***	0.8	1.7	1.1

***crp***	1.4	2.2	1.3

**Rd (CDM)**	4.8	3.8	2.1

Values given are for the ratio of the expression level of the gene following growth in BHI in the absence of added Neu5Ac to growth with added Neu5Ac, taken from the data given in Figure 6. Also shown are the values for strain Rd following growth on CDM medium. A dashed line indicates no expression following inactivation of the respective gene.

The most significant change in gene expression detected in a *crp *mutant in the Rd strain background was for the *siaP *gene, expression was decreased 19 fold when compared to the parent strain following growth in the absence of Neu5Ac (Figure 6). A similar reduction was observed following growth on both BHI and CDM media, although the magnitude of the change was less on CDM. No response to the presence or absence of Neu5Ac in the medium was observed for *siaP *expression in strain Rd*crp*. No significant change in either *nanE *or *siaR *expression was found in strain Rd*crp *with respect to the parent strain following growth on CDM, although an up to 4 fold change was observed using BHI medium (Figure 6). These data are consistent with the previous observation that CRP has a differential effect on sialometabolism genes, having a preferential role in activating uptake rather than catabolic genes [12].

*HI0148*, the gene downstream of *siaQ/M *encodes a protein that contains six Kelch motifs that are often associated with sialic acid binding proteins such as neuraminidase enzymes [30]. A Rd*HI0148 *mutant strain showed some loss of the lowest molecular weight glycoforms (Figure 2), but no difference in serum sensitivity (Figure 3), when compared to the wild type. No significant change in sialometabolism gene expression was observed following mutation of *HI0148 *(data not shown).

## Discussion

Sialic acids are a diverse family of sugars and are components of bacterial surface macromolecules such as capsular polysaccharides and glycolipids that are of major biological importance in pathogenesis. In *H. influenzae*, Neu5Ac is a potential carbon and energy source [8,12] as well as a component of the LPS of almost all NT*Hi *strains where detailed structure has been determined to date [26,31-33]. *H. influenzae *lacks the genes required for the synthesis of Neu5Ac and in nature must acquire it from humans, its only natural host. It has been shown that *H. influenzae *acquires Neu5Ac during experimental infection of chinchillas and that its incorporation into LPS is critical for virulence [3]. It has been estimated that the concentration of Neu5Ac potentially available in human tissues and fluids is 0.5 mg/ml [8] making it a potential major nutrient for the bacterium *in vivo*.

In the present study we have investigated genes involved in the dynamic interplay between utilisation of Neu5Ac in the biosynthesis of LPS (sialylation) or its potential as a catabolite. Microarray [25] and bioinformatic [8,12] analyses had identified a set of 9 contiguous genes that played a significant role in sialometabolism. We reasoned that an investigation of the transcription of *H. influenzae *sialometabolism genes would provide further insights into the genetic regulation relating to sialometabolism. Our study presents a number of novel or different findings from the study of Johnston and colleagues [12], including the effect of Neu5Ac in modulating transcription of sialometabolism genes, the conserved organisation of the sialometabolism genes, and the effects of mutation of the regulatory genes, *siaR *and *crp*, on experimental infection in a chinchilla animal model of OM.

The sialometabolism locus consists of nine genes, organized such that divergently transcribed catabolism and transport genes, are separated by an intergenic, non-coding region of 353 bp. This intergenic region contains a consensus CRP binding site and an overlapping site to which SiaR binds [12]. In the NT*Hi *strain (2019) studied by Johnston and colleagues, they conclude that exogenous Neu5Ac did not affect transcription of either the catabolic or transporter sialometabolism genes. In contrast, we have observed Neu5Ac-dependent transcriptional down-regulation when *H. influenzae *was grown in both BHI, a relatively complex medium, and CDM, a more defined medium. The transcriptional down-regulation of both transporter and catabolic genes that we had previously observed using DNA microarrays has now been confirmed and quantified by q-PCR. As an important indication of the general significance of sialometabolism to *H. influenzae *biology, the present study provides molecular epidemiological evidence that the sialometabolism gene cluster is conserved across a set of NT*Hi *strains that are representative of the genetic diversity found in the natural population of NT*Hi *[17]. This genetic conservation of sialometabolism genes between strains is in contrast to the well documented inter-strain LPS structural diversity that includes the variable location and stoichiometry of Neu5Ac, which is characteristic of NT*Hi *strains [26,33].

Sialometabolism genes are found clustered in many other bacterial species [9]. *siaR *homologues exist in other proteobacteria, e.g *Pasteurella sp*. but in the context of a different gene organisation [9]. In *Pasteurella multocida*, a pathogen of cattle and birds, the sialic acid TRAP transporter genes are located adjacent to catabolic genes that have a somewhat different gene organisation to *H. influenzae *[34].

Details of the mechanism(s) by which exogenous Neu5Ac alters transcriptional activity in *H. influenzae *remains unclear. Purified SiaR protein has been investigated by Johnston and colleagues [12] and has been demonstrated to bind to the intergenic region to down-regulate transcription of genes for the uptake and catabolism of sialic acid. Using RT-PCR and q-PCR in different strains of *H. influenzae*, we provide corroborating evidence that there is increased transcription of sialometabolism genes when *siaR *is disrupted. Mutation of *siaR *in our study resulted in up to a 19 fold increase in expression of sialometabolism genes tested. These changes are of a similar magnitude to the increased expression of the sialometabolism genes (range 2 to 16 fold) compared to the parent strain observed by Johnston and colleagues in a *siaR *mutant of NT*Hi *2019 [12]. A reasonable hypothesis is that the SIS domain [14] present in the SiaR protein could be a binding site for Neu5Ac, or perhaps other related sugars (e.g. N-acetylglucosamine or glucosamine-6-phosphate), that activate(s) the repressor activity of SiaR. Our findings from q-PCR provide clear evidence of a role for CRP as a positive transcriptional activator through its interaction with the consensus binding site located in the intergenic region in the middle of the sialometabolism genes, findings in agreement with previous studies [12]. In contrast to SiaR, which affects both catabolic and transporter genes, CRP is a positive transcriptional regulator primarily of Neu5Ac uptake.

An unexplained and intriguing aspect of sialometabolism in *H. influenzae *is the potential role for the HI0148 protein. The HI0148 protein contains Kelch motifs and recent studies in *E. coli *have shown that a homologue of the HI0148 protein, NanM, functions as a Neu5Ac mutarotase [35]. This mutarotase converts α-Neu5Ac to the β- form and vice versa. In solution, free Neu5Ac will tend to spontaneously shift towards the β-form. It is an interesting possibility that HI1048 could provide the correct anomer of Neu5Ac for uptake, or perhaps for catabolism or regulation. The function of NanM in *H. influenzae *is currently under investigation.

The crucial role of sialylation of LPS in the pathogenesis of *H. influenzae *infection has been demonstrated in a chinchilla model of OM [3]. Sialylation of NT*Hi *LPS interferes with the binding, activation and immune clearance of *H. influenzae *effected by complement components [5]. Mutant strains in which the Neu5Ac TRAP uptake system has been disrupted (e.g. *siaP *mutants) are deficient in LPS sialylation and we show here that these mutants are attenuated, although the degree of attenuation was greater for strains 486 and Rd than for 375. This finding emphasises the complexity of the mechanisms affecting host immune clearance but are broadly consistent with the relatively decreased LPS sialylation of strain 375 when compared to strain 486 [2]. Disruption of the TRAP transport system in *P. multocida *similarly attenuated bacterial virulence in the mouse [34] and turkey [36] models of systemic infection.

In contrast to the attenuation of *siaP *mutants in each of three *H. influenzae *strains tested, mutation of the genes encoding both the regulatory proteins SiaR and Crp showed no or little effect on virulence over the course of a 19 day infection in the chinchilla. We have shown that LPS remains sialylated in each of these mutant strains. Analysis of the sialylation profiles of the LPS isolated directly from bacteria taken from the middle ears of animals infected with these mutant strains could provide critical supportive *in vivo *evidence of LPS sialylation. Future studies should use an ascending model of infection in which infection is initiated through inoculation of the nasopharynx. The more relevant selection pressures contributing to the evolution of LPS sialylation and its regulation are likely to be a function of *H. influenzae *fitness for carriage and transmission rather than its role in disease. An understanding of the role of sialic acid, provided by the host, to the commensal and virulence lifestyles of *H. influenzae *would provide valuable insights into an aspect of host microbial interaction that might provide novel targets for intervention in disease caused by this bacterium.

## Conclusion

Expression of a set of genes required for sialometabolism in *H. influenzae *is altered through growth of the bacteria in the presence of sialic acid. Mutation of representative genes influences sialylation of the LPS molecule. The regulation of sialometabolism gene expression is complex but there appears to be no major requirement for the positive (CRP-dependent) or negative (SiaR-dependent) transcriptional regulation on LPS sialylation in experimental OM induced through direct inoculation of organisms into the middle ear of chinchillas.

## Authors' contributions

GAJ helped to design and carried out the transcription experiments, WAS analysed the combined data and helped to draft the manuscript, KM carried out the LPS gel and SBA analyses, GAK carried out the q-PCR analysis, MAF and SIP designed and carried out the chinchilla experiments and helped draft the manuscript, ERM and DWH conceived the study and helped analyse the data and draft the manuscript. All authors read and approved the final draft.
